# Rotation of a toric intraocular lens with and without capsular tension ring: data from a multicenter non-inferiority randomized clinical trial (RCT)

**DOI:** 10.1186/s12886-019-1147-5

**Published:** 2019-07-08

**Authors:** Ursula Hahn, Frank Krummenauer, Stefanie Schmickler, Jörg Koch

**Affiliations:** 1 Institute for Medical Biometry and Epidemiology, Witten/Herdecke University, Faculty of Health, Alfred-Herrhausen.Straße 50, 58448 Witten, Germany; 2 OcuNet Trial Alliance, Duesseldorf, Friedrichstraße 47, 40217 Duesseldorf, Germany; 3 Augen-Zentrum-Nordwest, Domhof 15, 48683 Ahaus, Germany; 4 Augenzentrum am St. Franziskus-Hospital, Muenster, Hohenzollernring 74, 48145 Muenster, Germany

**Keywords:** Toric intraocular lens, Rotation, Misalignment, Capsular tension ring

## Abstract

**Background:**

Evaluation of clinical outcome in cohorts with versus without simultaneous implantation of a capsular tension ring (CTR) and a toric lens (Tecnis Toric). Main parameter was rotation referring - in contrast to misalignment - to the IOL axis change from immediately after implantation to the final postoperative position.

**Methods:**

Lens position was measured at baseline with the patient still in recumbent position, postoperative rotation was calculated by software. Postoperative evaluation included measurement three months after surgery or prior to an indicated revision surgery. Explorative re-evaluation of the underlying RCT’s intent-to-treat population was performed for the entire sample and stratified for cohorts by 95% confidence intervals for binary endpoints’ incidences (primary endpoint: absolute postoperative rotation ≤5 degrees; secondary endpoints: absolute deviation between achieved cylinder and target cylinder ≤0.5 dpt, postoperative corrected distance visual acuity (CDVA) ≥ 0.8). Data exploration was based on medians and quartiles.

**Setting:**

Outpatient study sites.

**Design:**

Re-evaluation based on data from a multicenter non-inferiority randomized clinical trial (RCT).

**Results:**

Sub cohorts (without CTR 89, with CTR 90 patients) did not present clinically relevant differences in preoperative characteristics: revision surgery was performed in 7 cases (3 without and 4 with CTR). Primary endpoint incidences for the total sample, without and with CTR were 90%/89%/90%; cylinder endpoint incidences were 46%/45%/46% and CDVA endpoint incidences 90%/92%/88%. Median absolute rotations were 1.74°/1.79°/1.72°, median absolute cylinder deviations 0.55/0.52/0.55 dpt and median visual acuity 1.0/1.0/1.0.

**Conclusion:**

No clinically relevant differences between CTR subgroups were found; a satisfying three months rotational stability was achieved.

**Trial registration:**

The trial was registered retrospectively in the trial registry DRKS, trial registration number DRKS00015316, date of registration 27. August 2018.

## Background

The positional stability of a toric intraocular lens substantially determines the success of the refractive intervention. Deviations of postoperative position from target position (misalignment) occur either intraoperatively or after implantation. Misalignment is therefore caused by intraoperative deviation and/or positional change after implantation. Intraoperative deviation from target position are iatrogenic as a rule (induced by the physician) and therefore essentially influenceable. Positional change after implantation is called rotation and occurs beyond any immediate control.

There are many anecdotal reports suggesting that a capsular tension ring (CTR) might be helpful in reducing rotation of the toric lens following implantation. However no pertinent comparative data have been reported until now. One aim of the present prospective multicenter cohort study was therefore to quantify the postoperative rotation of a hydrophobic toric intraocular lens – in otherwise healthy eyes with cataract and regular astigmatism. The second idea was to identify possible differences in terms of rotation and other clinical outcomes after implant insertion between one cohort of patients who received a CTR and a toric lens, and a second cohort without CTR, and to record further clinical outcome parameters.

## Methods

Evaluated data are based on a multicenter randomized controlled trial; results of that multicenter non-inferiority study will be published elsewhere based on the per protocol population’s comparative findings. Patients with an indication for implantation of a toric lens were randomly assigned to a group either with (CTR group) or without CTR (non-CTR group); a net sample size of 96 patients per group was targeted.

Only one eye of each patient was included in the study; the study eye had to show regular astigmatism with an absolute corneal cylinder of 0.75 dpt to 2.75 dpt, and the lens to be implanted had to have a power of + 16 dpt to + 26 dpt. Patients were only included, if after surgeon’s estimation apostoperative corrected distance visual acuity (CDVA) of > 0.5 ought to be obtainable. Exclusion criteria were pre-existing conditions, such as endothelial dystrophy, pronounced cornea guttata, heavy corneal scars / clouding, glaucoma, uveitis, posterior synechiae, pseudoexfoliation syndrome, non-diabetic proliferative retinopathy stages II and III, proliferative diabetic retinopathy, condition following ocular trauma, age-related macular degeneration, or relevant previous ophthalmic surgery with either expected poor vision after surgery or instability of the capsular bag.

Data were collected pre-, intra- and postoperatively during the three months follow-up of this investigation. Regular postoperative data collection was performed 12 weeks after surgery. In cases requiring surgery to revise the lens position, data obtained immediately prior to revision surgery were included in the analysis.

For all cases in this study the hydrophobic acrylic lens Tecnis Toric 1-Piece ZCT (Johnson & Johnson Vision (JJV), formerly Abbott Medical Optics (AMO)) and the CTR Injector Ring by JJV were used. The lens position and power were calculated with the internet based Toric Calculator (Johnson & Johnson Vision). The calculation did not consider the posterior corneal astigmatism. Study sites were requested to employ the IOL formula SRK/T with an A-constant of 119.3.

In order to separately record rotation after implantation of the toric lens, the lens position was first measured (baseline) immediately upon completion of surgery with the patient still in a horizontal position. The second measurement was taken either at the regular follow-up three months after implantation, or prior to an indicated surgical repositioning, both in a vertical, sitting position. At baseline as well as postoperatively, the anterior segment was photographed in mydriasis, and the lens position was objectified via immovable landmarks of the iris and the sclera vessels ( [1], [2], [3], [4]). A proprietary tracking software (Johnson & Johnson Vision) was used to evaluate possible changes in the lens position.

### Choice of analysis population

The underlying Randomized Clinical Trial (RCT) was carried out according to the guidelines of “Good Clinical Practice”, in particular comprising strict raw data monitoring procedures as well as standardized data management and confirmatory statistical evaluation. As indicated above, this cohort data was derived from a confirmatory non-inferiority RCT; as the latter did not achieve the targeted sample size of 2 × 96 patients per group because of withdrawals the authors decided to perform an analysis of the available RCT sample in terms of a cohort evaluation. The analysis presented in this paper is based on an exploratory methodology. The presented results are therefore derived from an intent to treat population by means of a comparative analysis for superiority – in contrast to the initial (confirmatory) RCT analysis based on the per protocol population and a non-inferiority testing approach. To thoroughly admit this deviation from the intended RCT evaluation we emphasized the revised approach as exploratory. This cohort evaluation seeks to characterize the postoperative outcome of the cohorts with versus without CTR from a clinical perspective to provide clinical information about the expectable postoperative rotation.

During the study of the confirmatory non-inferiority RCT a large number of unexpected protocol violations was observed, in addition a notable difference in sample sizes among trial sites was found despite the intended underlying center-stratified randomisation scheme. As a consequence the per protocol population (as the indicated patient sample for a non-inferiority evaluation) turned out severely reduced in size and representativeness; important clinical information on adverse event profiles as well as on clinical interactions would no longer be available after restricting data evaluation to this per protocol sample. To retain this clinical information portfolio a secondary “intent to treat” evaluation was implemented to derive maximum clinical implication from the available patient data.

### Statistical methods

An explorative analysis of clinical characteristics across the entire intent-to-treat sample as well as stratified for the sub-cohorts with and without CTR was performed. The evaluation was based on the intent-to-treat population and thus considers a population other than that in the non-inferiority RCT; another difference is that the focus of this analysis is also on absolute postoperative rotation instead of primarily cylindric deviation as in the underlying RCT.

The re-evaluation of the underlying RCT’s intent-to-treat population was performed for the entire sample and stratified for cohorts by means of 95% confidence intervals for binary endpoints’ incidences (primary endpoint: absolute postoperative rotation <=5 degrees; secondary endpoints: absolute deviation between achieved cylinder and target cylinder <= 0.5 dpt, postoperative CDVA > = 0.8). Data exploration of the underlying continuous outcome characteristics was based on medians and quartiles for continuous parameters and on relative frequencies for categorical parameters, exploratory sub sample comparisons were based on two sample Wilcoxon and Chi2 tests (*p*-values were not formally adjusted for multiplicity according to the exploratory nature of the intent-to-treat Population’s evaluation).

## Results

Eleven study sites recruited a total of 188 patients between August 2014 and March 2016 (range per site between 3 and 37). Data from 179 study patients randomly assigned to the groups without (*n* = 90) and with CTR (*n* = 89) (intent-to-treat population, Table 1) were included in the subsequent explorative analysis: the CTR sub-samples did not present clinically relevant or statistically significant differences in terms of socio-demographic or ophthalmological parameters (Table 1). Furthermore, no tendencies of clinical relevance were documented between the CTR sub-samples with regard to width or type of incision (Table 2).

**Table 1:** Socio-demographic and ophthalmological characteristics

	total	without CTR (non-CTR sub sample)	with CTR (CTR sub sample)	p-value
total number of study eyes	179	90	89	
… per center	3–37	1–19	2–18	
age [years]	71.0 (61.8; 76.3)	71.0 (60.8; 76.0)	70.0 (61.3; 77)	0.718
female patients %	47%	44%	50%	0.431
CDVA (decimal)	0.5 (0.32; 0.5)	0.5 (0.32; 0.5)	0.4 (0.3; 0.5)	0.608
Axial length [mm]	23.8 (23.2; 24.4)	23.7 (23.2; 24.3)	23.9 (23.3; 24.6)	0.168
Cylinder [dpt] AR	−1.5 (−2.25; −1.0)	−1.5 (− 2.25; − 1.0)	−1.75 (− 2.63; − 1.0)	0.537
target cylinder. [dpt] Toric Calculator	− 0.1 (− 0.22; 0.03)	−0.08 (− 0.16; + 0.03)	−0.14 − 0.25; 0.06)	0.555
SEQ [dpt] AR	− 1.0 (− 3.13; + 0.88)	− 1.06 (− 2.91; 1.0)	1.0 (−3.56; 0.56)	0.356
target refraction. [dpt]	0.0 (− 0.13; 0.06)	0.0 (− 0.13; 0.06)	0.0 (− 0.13; 0.06)	0.631
IOL-M. K1. [mm]	7.84 (7.68; 8.01	7.82 (7.67; 7.97)	7.86 (7.69; 8.06)	0.141
IOL-M. K2. [mm]	7.55 (7.41; 7.72)	7.54 (7.39; 7.66)	7.58 (7.41; 7.74)	0.334
CT. K1. [mm]	7.84 (7.67; 8.01)	7.83 (7.7; 7.98)	7.88 (7.67; 8.04)	0.201
CT. K2. [mm]	7.56 (7.43; 7;72)	7.56 (7.39; 7.72)	7.60 (7.44; 7.72)	0.427

Pooled study sample (intent-to-treat population), stratified with and without capsular tension ring (CTR), medians and interquartile ranges, relative frequencies as well as nominal *p*-values derived from two-sample Wilcoxon tests and Chi2 tests (*p*-values not adjusted for multiplicity)

SEQ: Spherical Equivalent, IOL-M: IOL Master (Carl Zeiss Meditec), CT: Corneal Topography, CDVA corrected distance visual acuity, CTR: Capsular Tension Ring, AR: Auto Refractometer, K: Keratometry, mm: millimeter

**Table 2:** Intraoperative characteristics

	total	without CTR (non-CTR sub sample)	with CTR (CTR sub sample)	p-value
N=	179	90	89	
width of incision [mm], median, interquartile range	2.6 (2.4; 2.8)	2.6 (2.4; 2.8)	2.6 (2.45; 2.8)	0.931
Type of incision [%]				
• clear cornea • sclerocorneal • limbal	32% 30% 37%	33% 29% 38%	32% 32% 37%	

Pooled study sample (intent-to-treat population), stratified with and without capsular tension ring (CTR), medians and interquartile ranges, relative frequencies as well as a nominal p-value derived from a two-sample Wilcoxon test (p-value not adjusted for multiplicity)

CTR: Capsular Tension Ring

Postoperative data from 173 study patients were collected (non-CTR-group 84, CTR group 89), 7 data sets with reported revision surgery were included in the postoperative evaluation, whereby three of those belonged to the non-CTR-Group and four to the CTR-group.

Table 3 present postoperative findings for the pooled sample as well as stratified for the sub-samples with and without CTR. Incidences of the primary endpoint (postoperative absolute rotation <=5 degrees) were 89% in the non-CTR-group (95%-CI 81–96%) and 91% in the CTR-Group (95%-CI 84–98%) (Table 3). Data from 139 study eyes were available for the analysis of rotation (non-CTR-group 72, CTR-group 67), the data set contained only two photo documentations of lenses prior to revision surgery; rotation in these cases was + 38° and − 11°, respectively. Median absolute rotations of the underlying continuous outcome parameter were 1.80° in the non-CTR-group (Interquartile range 0.77°; 3.18°) and 1.72° in the CTR-group (Interquartile range 0.83°; 3.6°).

**Table 3:** Relative frequencies and 95%-confidence interval (95%-CI) relating to the primary and two secondary endpoints, medians and interquartil ranges for the underlying continuous outcome parameter

		without CTR (non-CTR sub sample)	with CTR (CTR sub sample)	total (pooled)
Primary endpoint: rotation				
n=		72	67	139
postoperative absolute rotation <= 5 degrees	incidence	89%	91%	90%
	95%-CI	81–96%	84–98%	85–95%
postoperative absolute rotation	median	1.787°	1.722°	1.737°
	Interquartile range	0.769°; 3.18°	0.829°; 3.589°	0.801°; 3.372°
Secondary endpoint: cylinder				
n=		84	85	169
absolute postoperative deviation of achieved cylinder from target cylinder ≤ ±0,5 dpt	incidence	45%	46%	46%
	95%-CI	34–56%	35–57%	38–54%
absolute postoperative deviation of achieved cylinder from target cylinder	median [dpt]	0.52	0.55	0.55
	Interquartile range [dpt]	0.38; 1.01	0.28; 0.93	0.34; 0.98
Secondary endpoint: BVCA				
n=		84	88	172
CDVA > = 0.8	incidence	92%	88%	90%
	95%-CI	86–98%	80–95%	85–94%
postoperative CDVA	median	1.0	1.0	1.0
	Interquartile range	0.8; 1.0	0.875; 1.0	0.8; 1.0

Pooled study sample (Intent-to-treat population), stratified with and without capsular tension ring (CRT)

CDVA: Corrected distance visual acuity

CTR: Capsular tension ring

CI: Confidence Interval

dpt: Dioptre

The secondary endpoint (postoperative absolute deviation of achieved cylinder from target cylinder ≤ ±0.5 dpt) was reached in 45% in the non-CTR eyes (95%-CI 34–56%), and in 46% in the CTR eyes (95%-CI 35–57%) (Table 3). The median postoperative residual cylinder was − 0.75 dpt for both groups, with an interquartile range of (− 1.0; − 0.5). The median absolute postoperative deviation of achieved from target cylinder was 0.52 dpt (interquartile range of 0.38; 1.01) for the non-CTR-eyes and 0.55 dpt (interquartile range of 0.28; 0.93) for the CTR eyes.

In the non-CTR sub-sample 92% of the study eyes (95%-CI: 86–98%) achieved the visual secondary endpoint compared to 88% (95%-CI: 80–95%) in the CTR sub-sample (Table 3). A median CDVA of 1.0 was found in both sub-samples.

Absolute deviation of the postoperative spherical equivalent (determined via auto refractometer) from the respective target cylinder was 0.35 dpt (interquartile range 0.17; 0.59) in the cohort without CTR versus 0.29 dpt (interquartile range 0.16; 0.5) in the CTR cohort.

## Discussion

The primary aim was to quantify rotation patterns after implantation of the study lens Tecnis Toric in patients with cataract and astigmatism in subgroups with and without simultaneous implementation of CTRs. Only healthy study eyes with lens powers ranging from + 16 dpt to + 26 dpt were included. Due to the recruiting criteria clincial and other refractive pathologies were not represented in the data set. In this study one cohort received the CTR with the toric implant, while the other cohort received only the toric implant. There were no significant clinical differences between the two sub-samples neither prior to surgery (Table 1) nor with regard to intraoperative characteristics (Table 2).

Overall good clinical results (Table 3) were achieved for the parameters rotation, refraction accuracy and visual acuity in the cumulative data set and in both cohorts with and without CTR according to binarized endpoints (with 95%-CI) and by means of median and interquartile ranges (Table 3). In both groups the median absolute rotation was less than 2 degrees, the median absolute deviation of achieved cylinder from target cylinder was lower than 0.55 dpt and the three months median CDVA was 1.0. The relative frequencies of the primary endpoint and both secondary endpoints were close with their respective nominal 95%-CI in large parts overlapping. Medians and interquartile ranges also showed a high degree of congruence between the non-CTR-group and the CTR-group across all three clinical characteristics.

### Rotation after implantation instead of total misalignment

The correct postoperative position of a toric lens is a crucial factor in successful astigmatism correction [5]: “A deviation of 10° will reduce the correctional effect by about one-third; a deviation of 20° can reduce the effect by about two-thirds. Postoperative astigmatism may increase when the deviation is 30°” [1]. A deviation of 5° is considered clinically relevant, and a deviation of 10° is deemed a borderline indication for revision surgery [6], [7].

Misalignment i.e. the entire deviation of realized lens position from target position [1] can have differente causes: Misalignment may result from intraoperative malpositioning [8], [9], [10]. Postoperative rotation is another partial quantity of misalignment, Whereas the causes of intraoperative malpositioning are known, iatrogenic as a rule and therefore basically avoidable (e.g. incorrect target axis or marking or alignment of the IOL, capsulorhexis tear), the surgeon has no influence on rotation to occur after implantation. This underlines the importance of specific research into factors which influence rotation. Influencing factors currently under debate are size of the capsular bag and asymmetric capsular shrinkage [11], but also properties of the lens.

Table 4 gives a comprehensive overview of values for misalignment and rotation of the study lens Tecnis Toric as reported in the literature and determined in the context of this study. Intraoperative malpositioning may be determined as difference between misalignment and rotation, and is not explicitly reported in pertinent publications. Mean values for misalignment or rotation are generally reported as absolute values in the literature. Table 4 indicates literature sources where the wording is ambiguous in this respect – a higher absolute rotation must be assumed in cases where original values with positive and negative signs were included in the calculation of the median. The misalignment mean values of the study lens ranged from 2.48° [2] to 7.67° [3]; the misalignment of the study lens largely corresponds to values for other lenses reported in the literature [4], [5], [12]. Mean values for rotation ranged from 2.2° [6] to 2.7° [8], rotation in the entire population of the study under investigation was 2.7 °, in the sub-group 2.7° (without CTR) and 2.8 ° (with CTR). The mean value for rotation in the few available data sources fell below the threshold of 3° which is deemed to be the limit of perception for changes in position.

**Table 4:** Overview of study results reported in the literature on misalignment and rotation of the toric lens Tecnis Toric with mean values and standard deviations

First author	Year of publication	No. of eyes	Follow-up post-op	Misalignment (abs)*	Range Misalignment (abs)	Rotation after implantation (abs)*	Baseline	Range Rotation
Hirnschall [6]	2014	30	3 mo	3.6° ±3.2°	Max 13.9°	2.2° ±3.1	“1 h after surgery”	≤ 3° 59%
								≤ 6° 91%
Waltz [8]	2015	156	6 mo			2.7 ± 5.5	“1 day after surgery”	≤ 5° 94%
								≤ 10° 97%
OcuNet, entire population*	2017	139	3 mo/prior to Re-OP			2.7 ± 3.9	Immediately after surgery. Patient still recumbent	≤ 3° 68%
								≤ 5° 90%
								≤ 6° 91%
								≤ 10° 96%
OcuNet, without CTR*	2017	72	3 mo/prior to Re-OP			2.7 ± 2.8		≤ 3° 74%
								≤ 5° 89%
								≤ 6° 90%
								≤ 10° 97%
OcuNet, with CTR*	2017	67	3 mo/prior to Re-OP			2.8 ± 4.7		≤ 3° 63%
								≤ 5° 91%
								≤ 6° 91%
								≤ 10° 94%
Ferreira [24]	2012	20	2 mo	3.15° ±2.62°a	no eye > 10°			
Mazzini [25]	2013	19	6 mo	3.33° ± 1.94°	1° - 10°			
Sheppard [26]	2013	61	4–8 we	3.4°	0° to 12 ° 9 eyes (15%) > 5° 1 eye > 10°			
Dominguez [27]	2014	53	3 mo	3.1° ±2.8°b	0° - 12°.			
Jacobs [28]	2015	22	Median: 11.3 mo	7.5° c	0° - 21°. in 31.8% > 10°			
Elhofi [2]	2015	Group 1: 30 Group 2: 30	3–5 we	Group 1: 2.48° ±1.96° Group 2: 4.33° ±2.72° d	Group 1: 0° - 7 Group 2: 1° - 12°			
Lam [29]**	2016	31	3 mo	7.67° ±4.04°e	3 eyes “with misalignment”			
Grohlich [30]**	2017	n. r.	12–36 mo	4.31° ±4.59° f	4 eyes with rotation > 10°			
Mies [31]**	2017	88	2 mo	Median 3.0° ±4.46° g	9 of 67 eyes. Rotation > 10°			

* In contrast to Table 3, Table 4 refers to the mean value / standard deviation in the entire population (patients at regular follow-up or prior to possible revision surgery)

** Literature source does not state unambiguously whether misalignment was calculated from original values with positive and negative signs or from absolute values

n. r.: not reported, CTR: capsular tension ring, mo: months, we: weeks, post-op.: postoperative, Re-OP: revision surgery

Only one study reports misalignment as well as rotation [1]; intraoperative malpositioning of 1,2° on average may be deduced from the difference (Table 3). The result suggests that postoperative rotation has a larger share in lens misalignment than intraoperative malpositioning. These findings underline the need to explore rotation, its causes and, above all, options to limit rotation.

Earlier studies and case reports indicate that rotation may occur in the early stages after implantation, merely lifting the patient from the horizontal position may already cause further change in lens position. [9], [10], [11]. According to a recently published work rotation was significantly increased within the first hour after operation compared with later time-points (*p* < 0.001) [7]. Initial measurement (baseline) must take place with the patient still in a recumbent position so that potential malpositioning of the IOL induced by the surgeon may be clearly distinguished from subsequent rotation. Procedures used for the study followed those described by Weinand et al. for a non-toric lens [3]. Descriptions of measurement points in both literature sources (cf. Table 4: “one hour after surgery” [1], “one day after surgery” [4]) suggest a later time of measurement. Consequently, the possibility that the reported results fall short of actual rotation cannot be excluded.

Apart from the median (respectively, mean value) of rotation, the frequency of outliers in positional change is a further criterion in assessing the quality of refractive surgery. In the following part the authors discuss data set frequencies as collected in this study within the ranges used in the literature. The present study reported a rotation of ≤ ±6° for 91% of data sets for which it was possible to determine rotation; this corresponds to values published elsewhere for the Tecnis Toric [1] (Table 4). The proportion of data sets with a rotation of ≤ ±10° was 96%, another study reported a relative frequency of 97% [4]. Thus, less than one out of twenty patients required revision surgery merely as a consequence of rotation (without consideration of possible intraoperative malpositioning), provided a positional change of 10° and more is accepted as indication for revision surgery. Hirnschall et al. reported a maximum outlier of − 13,0° [1], whereas Waltz [8] did not mention any maximum values. The maximum value of an outlier found in this study was + 38.1°, the next was − 13.1°. Overall, the number of outliers is too small to indicate a trend, and rather to be understood as single case histories.

### Capsular tension ring (CTR) and rotation of toric lens

CTRs are discussed as an approach to avoid or reduce rotation. An important aim of the study was therefore to describe postoperative rotation in the entire data set as well as in both groups – with and without CTR implantation. It is undisputed that CTRs are indicated for deficiencies or dehiscence of the zonula ciliaris: they are implanted with the intention to avoid decentration and tilting of the IOL in pseudoexfoliation syndrome, high myopia and subluxated lenses [13]. The question as to whether and to which extent a CTR additionally effects rotation of a toric intraocular lens – under the condition of astigmatism in healthy eyes – is a subject of current scientific debate [14], [15], [16]. It appears plausible, for example, that a CTR positively impacts positional stability after implantation because it serves to fill the capsular bag better and to fix the loops.

To the authors’ knowledge, very few study findings on the subject matter have been published to date; previous publications were either case histories [17], [18], focussed on the axial lens position [19] or examined a cohort of patients with pre-existing high myopia [12]. Recently published results from a prospective randomized clinical trial performed in a tertiary care centre in India with a case number of 50 eyes (Auroflex Toric IOL) reported a statistically significant difference in mean rotation at 3 months in favour for the group with CTR (group with CTR: 1.85 ± 1.72°; group without CTR: 4.02 ± 2.04°) [20]. Another recent publication based on a cohort study of 34 patients (16 patients each in the CTR and the non-CTR group) showed statistically significant difference in UCVA between the two groups and more cases with IOL rotation (12 eyes) in the non-CTR group than in the combined group (4 eyes). In contrast to the CTR group there was a significant difference in the Non-CTR group between residual and predicted astigmatism [21].

In contrast to the findings in these two studies, the present study did not reveal neither clinically relevant nor statistically significant differences for rotation (or any other considered outcome parameters) between the patient cohorts with and without CTR; results of binary and underlying continuous endpoint evaluation in both sub-samples corresponded to a large extent. Results suggest that the simultaneous implantation of a CTR has no attributable impact on positional stability after implantation. However, it should be noted that this conclusion applies only to the hydrophobic lens and the specific CTR used in the study. It is conceivable that the impact of the CTR may produce different results if used in combination with other lenses with properties that facilitate rotation [22], [23] – such as hydrophilic material, different loop design or smaller IOL diameter.

### Limitations of data quality

Study data were subject to a number of limitations. Recruitment of patients for the study turned out to be more time consuming than expected. Recruitment for the randomized study had to terminate before the scheduled number of cases had been reached, since the medical products stipulated for this study (CTR, specific lens powers) were no longer available on the market. Moreover, success of recruitment varied considerably among the 11 study sites involved. The sites contributed between 3 and 37 patients to the study population. Data were incomplete in some cases, so that less data were available for the analysis of individual parameters.

The number of informative data sets differed significantly depending on the respective outcome parameter under consideration (Tables 3). In particular, five from seven data sets with a need for corrective surgery did not contain details on rotation. Photo documentation in mydriasis to determine rotation, to be performed immediately after surgery with the patient still in a recumbent position and postoperatively (at regular follow-up or prior to revision surgery), does not usually form part of regular treatment.

Gaps in documentation may therefore, at least to a certain extent, be attributed to the fact that the study was carried out in a commonplace care environment. Part of the existing photo documentation turned out to be not analysable at one or both measurement points: conditions were not fulfilled to ensure exact determination of position. These deficits did not become apparent prior to the reader stage and thus were irreversible. As a result, it cannot be ruled out that the rotation determined in this study may be too small or too high. But even with highly stringent research management along the lines of good clinical practice it was found almost inevitable that this pragmatic multicenter trial does not yield the degree of data quality expected and hoped for in the planning stage.

These limitations implied a number of formal and biasing protocol violations. Furthermore the sample size for the initial planed non-inferiority RCT study was not accomplished. As a consequence, the authors sought to present maximum available information on rotation patterns by re-evaluation of the initial RCT sample based on the intent-to-treat population. Change of data analysis focused on the three months follow-up of the non-CTR and CTR (intent-to-treat) sub-samples. Although this change of analysis population can be discussed critically from the formal regulatory perspective, the available data was presented to retain clinical and pragmatic “real world outcome patterns”.

## Conclusion

Rotation of the toric lens Tecnis Toric after implantation was quantified in a multicenter cohort study. In order to evaluate the potential of a CTR to avoid or limit postoperative rotation, one patient cohort was implanted simultaneously with a CTR whereas the other cohort received only the toric lens. Good results were achieved with reference to rotation after surgery, deviation of achieved cylinder from target cylinder and CDVA. The relative frequencies in the non-CTR-group und the CTR-group relating to the primary endpoint (postoperative absolute rotation <= 5 degrees) and two secondary endpoints (absolute postoperative deviation of achieved cylinder from target cylinder ≤ ±0,5 dpt; postoperative CDVA > = 0.8) were close, the 95%-CI in large parts overlapping. Medians and interquartile ranges were reported in addition and revealed no clinically relevant deviations.

The focus in this study was on postoperative rotation as a partial quantity of misalignment. The impact of intraoperative malpositioning on lens position was not investigated. Postoperative rotation is primarily due to causes that are beyond the surgeon’s control. It is therefore all the more important to evaluate the degree of, and approaches to limit, rotation. Rotation measured in the context of the study was below the clinically relevant limit of 3° for the entire population as well as for both cohorts respectively; differences between cohorts were not statistically significant. Expectations that the CTR can limit postoperative rotation or avoid outliers have not been confirmed for the hydrophobic lens used in this study which anyhow showed a very rotation.

## Data Availability

The Datasets generated and analysed during the current study are not publicly available due to further publications are under way but are available from the corresponding author on reasonable request.

## Abbreviations

CDVA: Corrected distance visual acuity
CI: Confidence interval
CTR: Capsular tension ring
IOL: Intraocular lens
RCT: Randomized clinical trial
